# Longitudinal impact of changes in the residential built environment on physical activity: findings from the ENABLE London cohort study

**DOI:** 10.1186/s12966-020-01003-9

**Published:** 2020-08-01

**Authors:** Christelle Clary, Daniel Lewis, Elizabeth Limb, Claire M. Nightingale, Bina Ram, Angie S. Page, Ashley R. Cooper, Anne Ellaway, Billie Giles-Corti, Peter H. Whincup, Alicja R. Rudnicka, Derek G. Cook, Christopher G. Owen, Steven Cummins

**Affiliations:** 1 Department of Public Health, Environments and Society, London School of Hygiene and Tropical Medicine, London, UK; 2 Population Health Research Institute, St George’s, University of London, London, UK; 3 Centre for Exercise, Nutrition and Health Sciences, University of Bristol, Bristol, UK; 4 National Institute for Health Research Bristol Biomedical Research Centre, University Hospitals Bristol NHS Foundation Trust and University of Bristol, Bristol, UK; 5 MRC/CSO Social and Public Health Sciences Unit, University of Glasgow, Glasgow, UK; 6 NHMRC Centre of Research Excellence in Healthy Liveable Communities, RMIT University, Melbourne, Victoria Australia

**Keywords:** Longitudinal, Built environment, Physical activity, Social inequalities, Neighbourhood walkability, Park proximity, Public transport accessibility, Steps, MVPA

## Abstract

**Background:**

Previous research has reported associations between features of the residential built environment and physical activity but these studies have mainly been cross-sectional, limiting inference. This paper examines whether changes in a range of residential built environment features are associated with changes in measures of physical activity in adults. It also explores whether observed effects are moderated by socio-economic status.

**Methods:**

Data from the Examining Neighbourhood Activity in Built Living Environments in London (ENABLE London) study were used. A cohort of 1278 adults seeking to move into social, intermediate, and market-rent East Village accommodation was recruited in 2013–2015, and followed up after 2 years. Accelerometer-derived steps (primary outcome), and GIS-derived measures of residential walkability, park proximity and public transport accessibility were obtained both at baseline and follow-up. Daily steps at follow-up were regressed on daily steps at baseline, change in built environment exposures and confounding variables using multilevel linear regression to assess if changes in neighbourhood walkability, park proximity and public transport accessibility were associated with changes in daily steps. We also explored whether observed effects were moderated by housing tenure as a marker of socio-economic status.

**Results:**

Between baseline and follow-up, participants experienced a 1.4 unit (95%CI 1.2,1.6) increase in neighbourhood walkability; a 270 m (95%CI 232,307) decrease in distance to their nearest park; and a 0.7 point (95% CI 0.6,0.9) increase in accessibility to public transport. A 1 s.d. increase in neighbourhood walkability was associated with an increase of 302 (95%CI 110,494) daily steps. A 1 s.d. increase in accessibility to public transport was not associated with any change in steps overall, but was associated with a decrease in daily steps amongst social housing seekers (− 295 steps (95%CI − 595, 3), and an increase in daily steps for market-rent housing seekers (410 95%CI -191, 1010) (*P*-value for effect modification = 0.03).

**Conclusion:**

Targeted changes in the residential built environment may result in increases in physical activity levels. However, the effect of improved accessibility to public transport may not be equitable, showing greater benefit to the more advantaged.

## Background

Physical inactivity is associated with a wide range of chronic illnesses including cardiovascular diseases, stroke, obesity and diabetes [1]. In high-income nations [2], including the UK [3], current population levels of physical activity (PA) are too low. In the past decade, research interest in the socio-ecological determinants of PA has grown [4], partly encouraged by the failure of individual level risk factors to fully explain differences in PA. Of particular interest has been the extent to which the neighbourhood residential environment shapes PA behaviours [5]. Factors such as neighbourhood walkability (e.g. [6, 7]), residential density (e.g. [7, 8]), land-use mix (e.g. [7, 9]), street connectivity (e.g. [7, 9, 10]), and accessibility to greenspace (e.g. [8, 11]) and to public transportation (e.g. [9, 11]) have been found to be associated with PA in the UK and elsewhere. However, most of these studies rely on cross-sectional designs, making them vulnerable to biases such as residential self-selection [12]. As environments can change in response to residents’ preferences, and residents may choose to live in locations consistent with their preferred lifestyles, cross-sectional designs limit our ability to make causal inferences [12]. Longitudinal studies that examine associations between time-varying features of the residential built environment and PA, for instance using natural experiments [13], have been proposed as one way of strengthening the evidence base for causal effects.

In this paper, we use longitudinal data from the Examining Neighbourhood Activities in Built Living Environments in London (ENABLE London) study to investigate whether changes in exposure to features of the residential built environment are associated with changes in objectively measured levels of PA among adult participants, and whether any observed effects are moderated by a marker of socio-economic status (aspirational housing tenure). The ENABLE London study capitalised on the rapid creation and occupancy of East Village (formerly the London 2012 Olympic and Paralympic Athletes’ Village, London (UK)), a purpose-built mixed-use residential development specifically designed to encourage healthy active living [14]. Adults seeking to move into different tenured accommodation, social housing (public housing provided by East Thames Group Housing Association), intermediate housing (a mixture of shared ownership, shared equity and affordable rent, managed by Triathlon Homes) or market-rent accommodation (owned by Get Living London) in East Village were enrolled in ENABLE London. They were followed-up after 2 years, once half had relocated to East Village [14]. Previous work on the ENABLE London cohort exploring the *group-level* intervention effect of relocating to East Village found that moving to East Village was associated with a small non-significant increase in mean daily steps (154, 95% CI -231, 539), whilst no effects were observed for other PA and health related outcomes [15]. The study presented here instead explores whether changes in *individual-level* exposures across the entire cohort are associated with changes in PA, allowing exploration of heterogeneity in changes in individual exposures. Specifically, we estimated within-person changes in GIS-derived neighbourhood walkability, residential density, land use mix, street connectivity, proximity to parks and accessibility to public transport, and examined their impact on accelerometer-derived daily steps taken and daily Moderate to Vigorous Physical Activity (MVPA) accumulated (minutes) at follow-up, while controlling for baseline PA; by controlling for baseline PA we are in effect looking at how changes in the built environment influence change in PA. We further explored whether these effects differed by housing tenure being sought (social, intermediate, market-rent), as a marker of socio-economic status.

## Methods

### ENABLE London study participants

Details of the ENABLE London study design and recruitment process have been described elsewhere [14]. The baseline sample consisted of participants aged 16 years and over seeking relocation into either a social, intermediate (a mixture of shared ownership, shared equity and affordable rent) or market-rent accommodation in East Village. East Village is a 67-acre site with over 6000 current residents and 35 acres of open land and parkland, a school, three playgrounds and several retail areas. Baseline assessments were carried out between January 2013 and January 2016 prior to any potential move to East Village. Participants were followed-up at 2 years between February 2015 and October 2017 after half of the participants had relocated to East Village, and the other half either moved elsewhere or remained at their address. Assessments at both baseline and follow-up took place at the participant’s place of residence (or at an agreed location), first among social, then intermediate, and finally market-rent seekers, as dictated by the order of availability of the accommodations in East Village. A team of trained fieldworkers administered self-completion questionnaires including questions about participants’ sociodemographic characteristics. Participants were also asked to wear a hip-mounted accelerometer (ActiGraph GT3X+) for 7 days. Full ethical approval was obtained from the relevant Multi-Centre Research Ethics Committee (REC Reference 12/LO/1031). All participants provided written informed consent.

### Variables

#### Accelerometer-derived physical activity outcomes

Mean daily steps taken and mean daily time (minutes) accumulated in MVPA (≥1952 counts per min [16]) were derived both at baseline and follow-up. Periods of time in which the accelerometers were not worn were defined as 60 min or more of zero values, allowing for a 2-min spike tolerance, to provide the daily wear time. Days of accelerometer data in which the duration of registered wear time accumulated was less than 540 min were excluded. Participants with at least 1 day of data at both baseline and follow-up were included in analyses (for more detail [17]). Daily steps and MVPA were adjusted for day of the week, day order of wear and month of wear.

### Environmental variables

Participants were geocoded to the centroid of the footprint of their building of residence at both baseline and follow-up using Ordnance Survey (OS) AddressBase Premium versions 2015 and 2017, respectively.

- Neighbourhood walkability

Street connectivity, land use mix, and residential density in participants’ residential neighbourhood [9, 18] were derived within a 1 km-street network home-centred buffer both at baseline and follow-up (see Supplemental material 1). Baseline and follow-up metrics were then converted to z-scores based upon the baseline sample mean and standard deviation [8]. Rescaling scores at follow-up using baseline population estimates ensured comparability of scores both across participants and across time for the same participant, hence providing a meaningful quantification of the change in walkability over time. Neighbourhood walkability was derived at baseline and at follow-up by summing the three baseline and the three follow-up z-transformed variables, respectively.

b) Proximity to a park

Using data from Greenspace Information for Greater London (GiGL) 2015 [19], a park proximity variable was computed at both baseline and follow-up. This was calculated as the shortest street-network distance from the residential address to the nearest entrance of the closest park. Park referred to either a metropolitan, district or local park as defined by the Greater London Authority (GLA) London Plan March 2016 [20]. Where there were missing entrance points to parks in the GiGL database (*n* = 22, i.e. 2.9%), they were manually geocoded based on visual inspection from Google Maps.

c) Public transport accessibility

Each ENABLE London participant was assigned a PTAL (Public Transport Accessibility Level) score based on the closest location to their place of residence where a PTAL value was made available by Transport for London (TfL) [21]. PTAL is a commonly used [22] averaged measure of the densities of the London public transport access points (trains, buses, underground, Docklands Light Railway (DLR), trams), that also accounts for frequency of service [23]. It is classified into six-value ranges (0, 1a, 1b, 2, 3, 4, 5, 6a, 6b: lower scores reflecting poorer accessibility), whose scores are available for the centroid of each 100 m by 100 m cell of a grid covering the whole of Greater London [23].

.Changes in neighbourhood walkability, distance to park, and public transport accessibility were calculated by subtracting the value at baseline from the value at follow-up.

Data sources and versions used for computing all these environmental variables are detailed in Supplemental material 2.

#### Covariates

Covariates included sex (female, male), age group (16–24, 25–34, 35–49, and 50+ years), ethnic group (White, Black, Asian, Mixed/Other), and aspirational housing tenure (social, intermediate, market-rent). As justified elsewhere [17], aspirational housing tenure was used as a proxy for socio-economic status, with “social” and “market-rent” referring to the most deprived and affluent groups, respectively.

### Statistical analyses

First, changes in neighbourhood walkability, residential density, land use mix, street connectivity, distance to park, and accessibility to public transport were quantified in the whole sample and by aspirational housing tenure. Second, multilevel linear regression models including a random effect to allow for clustering at household level were fitted using the MIXED command in Stata/SE 15 to examine the effect of changes in exposure to residential built environment features on changes in total daily steps and total daily MVPA (min) (one model per residential built environment exposure variable and per PA outcome). Rather than regressing change in physical activity on change in built environment and other covariates, we regressed physical activity at follow up on physical activity at baseline as well as other covariates. Average daily steps (daily MVPA) at follow-up were thus regressed on average daily steps (daily MVPA) at baseline, adjusting for change in exposure as a fixed effect and household as a random effect. Models with further adjustment for sex, age group, ethnic group, and housing tenure (all measured at baseline) were also fitted. Finally, an interaction term between each change in environmental exposure (taken in turn) and housing tenure was included to test for effect modification. Sensitivity analyses further explored whether these effects differed by weekdays versus weekend days. All analyses were carried out using STATA/SE software (Stata/SE 15 for Windows; StataCorp LP, College Station, TX, USA).

## Results

Among the 1278 participants enrolled at baseline, 877 participants were followed-up at 2 years (response rate: 69%). Of those 877 followed-up participants, we excluded those who lived outside Greater London and those who did not have (enough) PA data at either baseline or follow-up (*n* = 190). Those excluded from the analytical sample had similar characteristics to those included with regard to sex and ethnicity, but had a slightly different age structure (*p* = 0.009) and included fewer intermediate and more market-rent seekers compared to those included (*p* = 0.003) (Supplemental material 3). Of the 687 participants retained for analyses, 283 were seeking relocation into social, 301 into intermediate, and 103 into market-rent accommodations.

Baseline descriptive statistics for the analytical sample are shown in Table 1. Women, middle aged (35–49 years) and those belonging to ethnic minorities were more prevalent among social compared with intermediate housing seekers (*p* < 0.001). Compared with participants seeking intermediate and market-rent housing relocation, social housing seekers were less physically active at baseline (social: 8162 steps and 54 min MVPA vs intermediate: 9458 steps and 63 min MVPA vs market-rent: 9611 steps and 67 min MVPA). Socio-demographic characteristics and levels of PA of the intermediate and market-rent housing seekers were largely similar. At baseline, social housing seekers resided in less walkable areas (walkability score social: -0.4, 95%CI − 0.6 to − 0.1; intermediate: 0.2, 95%CI − 0.1 to 0.5; market-rent 0.4, 95%CI − 0.2 to 1.1, *p* = 0.004), and had reduced accessibility to public transport (PTAL score social: 4.3, 95%CI 4.1 to 4.5; intermediate: 4.8, 95%CI 4.6 to 5.0; market-rent 5.0, 95%CI 4.6 to 5.3, *p*-value < 0.001).

**Table 1:** Baseline characteristics of the followed ENABLE London participants, by aspirational housing tenure and combined

	Total		Housing group						*p*-value for difference between housing groups
	*n* = 687		Social *n* = 283		Intermediate *n* = 301		Market-rent *n* = 103		
**Sociodemographics at baseline, n (%)**									
*Sex*									
Female	401	(58%)	206	(73%)	151	(50%)	45	(44%)	< 0.001^a^
*Age groups*									
16–24 years	130	(19%)	53	(19%)	50	(17%)	27	(26%)	< 0.001^a^
25–34 years	289	(42%)	72	(26%)	172	(57%)	45	(44%)	
35–49 years	223	(33%)	137	(48%)	70	(23%)	16	(16%)	
50+ years	45	(7%)	21	(7%)	9	(3%)	15	(15%)	
*Ethnicity*									
White	334	(49%)	52	(18%)	209	(69%)	73	(71%)	< 0.001^a^
Black	172	(25%)	135	(48%)	30	(10%)	7	(7%)	
Asian	110	(16%)	59	(21%)	43	(14%)	8	(8%)	
Mixed/Other	71	(10%)	37	(13%)	19	(6%)	15	(15%)	
**Residential built environment factors at baseline, mean (95%CI)**									
Walkability (score)	0.0	(−0.2;0.2)	-0.4	(− 0.6;-0.1)	0.2	(− 0.1;0.5)	0.4	(− 0.2;1.1)	0.004 ^b^
Connectivity (nb intersections/km of road)	8.6	(8.6;8.7)	8.5	(8.4;8.7)	8.7	(8.6;8.9)	8.8	(8.5;9.1)	0.06^b^
Residential density (1000hab/km2)	11.9	(11.5;12.3)	10.3	(9.8;10.8)	12.7	(12.0;13.4)	13.7	(12.4;15.1)	< 0.001^c^
Land Use Mix (score)	0.37	(0.36;0.39)	0.35	(0.33;0.37)	0.38	(0.36;0.40)	0.42	(0.38;0.46)	0.01^c^
Distance to the closest park (m)	663	(633;692)	609	(570;648)	703	(656;749)	694	(602;787)	0.06^c^
Accessibility to public transport (PTAL score)	4.6	(4.5;4.8)	4.3	(4.1;4.5)	4.8	(4.6;5.0)	5.0	(4.6;5.3)	< 0.001^b^
**Physical activity at baseline, Mean (95%CI)**									
Daily steps ^d^	8947	(8713;9182)	8162	(7742;8582)	9458	(9077;9840)	9611	(8984;10,238)	< 0.001^e^/0.67^f^
Daily minutes of MVPA ^d^	60	(58;61)	54	(50;57)	63	(60;66)	67	(62;72)	< 0.001^e^/0.12^f^

^a^ Chi-square

^b^ Anova

^c^ kwallis

^d^ Means are adjusted for sex, age group, ethnic group, housing sector and a random effect to allow for clustering at household level

^e^ t-test for the difference between “Social” and “Intermediate”

^f^ t-test for the difference between “Market-rent” and “Intermediate”

### Baseline to follow-up changes in exposure

Within-person changes in exposure to built environment factors over the two-year period between baseline and follow-up are shown in Table 2. Follow-up participants experienced a positive change in neighbourhood walkability of 1.4 units (95%CI 1.2 to 1.6). Social housing seekers had the greatest improvement in neighbourhood walkability (1.7 units, 95%CI 1.4 to 2.0) compared with intermediate (1.3 units, 95%CI 0.9 to 1.6) and market-rent (1.0 units, 95%CI 0.3 to 1.7) housing seekers; these differences were statistically significant across housing groups. Improvement in walkability scores was mostly driven by increases in residential density (7779 residential units/km^2^, 95%CI 6910 to 8648) and Land Use Mix (0.21 units, 95%CI 0.19 to 0.23). Participants experienced a mean decrease in distance to the nearest park of 270 m (95%CI 232 to 307), with no significant differences across housing groups. They also had a positive change of 0.7 units (95%CI 0.6 to 0.9) in accessibility to public transport, with social housing seekers experiencing the greatest amount of change (1.5 units, 95%CI 1.2 to 1.8) compared with intermediate (0.2, 95%CI 0.0 to 0.5) and market-rent housing seekers (0.1, 95%CI − 0.3 to 0.5); the *p*-value for difference across groups was highly significant.

**Table 2:** Within-person change (baseline to follow-up) in residential built environment characteristics overall and by aspirational housing tenure

	Total		Aspirational housing tenure						ANOVA *p*-value for differences between housing groups
	n = 687		Social n = 283		Intermediate n = 301		Market-rent n = 103		
*Changes in Built Environment Exposures*	Mean	(95%CI)	Mean	(95%CI)	Mean	(95%CI)	Mean	(95%CI)	
Change in walkability (score)	1.4	(1.2;1.6)	1.7	(1.4;2.0)	1.3	(0.9;1.6)	1.0	(0.3;1.7)	0.04
Change in connectivity (nb intersections/km of road)	−0.5	(−0.6;-0.4)	−0.6	(−0.7;-0.4)	−0.5	(− 0.7;−0.4)	-0.4	(− 0.7;-0.1)	0.54
Change in residential density (hab/km2)	7779	(6910;8648)	8902	(7608;10,197)	7197	(5866;8527)	6394	(3953;8835)	0.09
Change in Land Use Mix (score)	0.21	(0.19;0.23)	0.25	(0.21;0.27)	0.20	(0.17;0.23)	0.12	(0.07;0.17)	< 0.001
Change in distance to the closest park (m)	−270	(−307;-232)	− 303	(− 349;-256)	− 262	(−322;-203)	−201	(− 325;-76)	0.12
Change in accessibility to public transport	0.7	(0.6;0.9)	1.5	(1.2;1.8)	0.2	(0.0;0.5)	0.1	(− 0.3;0.5)	< 0.001

**Fig. 1:**
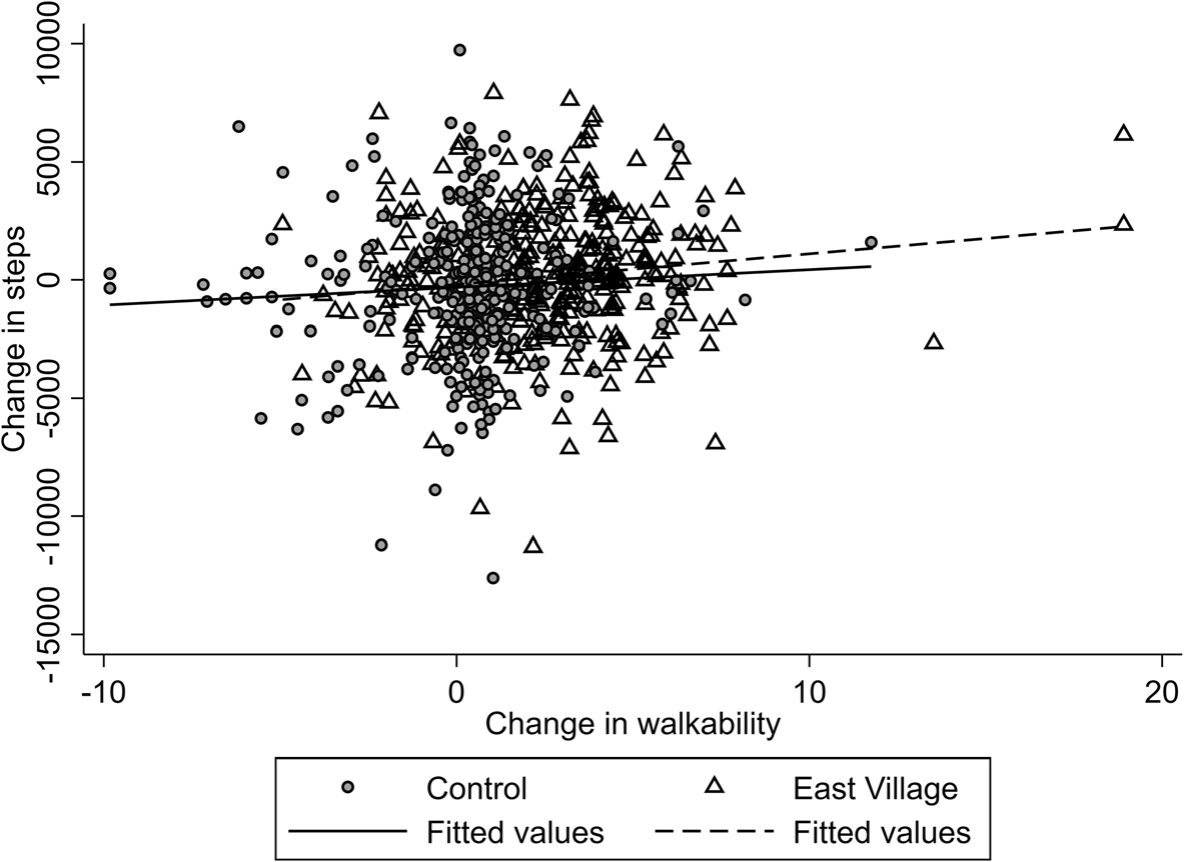
Change in steps and change in walkability, baseline to follow-up, by movers to East Village and non-movers to East Village

At follow-up, overall positive changes in exposure were primarily observed in the group of participants who relocated to East Village (*n* = 357) (see Supplemental material 4). They experienced a 2.4 units (95%CI 2.1 to 2.7) increase in neighbourhood walkability, a 531 m (95%CI 488 to 574) decrease in the distance to their nearest park, and a 1.6 points (95%CI 1.3 to 1.9) increase in accessibility to public transport. In contrast, near null *average* changes in these exposures (i.e. 0.3 units (95%CI 0.1 to 0.6) increase in walkability, 13 m (95%CI − 32 to 59) decrease in the distance to the nearest park, and 0.2 points (95%CI − 0.4 to 0.0) decrease in accessibility to public transport) were observed for the group of participants who did not move to East Village (*n* = 330) (see Supplemental material 4), regardless of whether they relocated elsewhere than East Village (*n* = 161) or remained at the same address (*n* = 169) (see Supplemental material 4). While a marked average improvement was seen in those moving to East Village, it is also notable that the individual changes varied considerably around this average – illustrated for walkability in Fig. 1. Despite the null average change in those not moving to East Village (Supplemental material 4), there was again considerable variability; while for those not moving walkability was effectively unchanged, among those moving, both positive and negative changes took place in the non-East Village group; presumably due to the variety of places moved to (see Supplemental material 4). The lines of best fit amongst those moving to East Village and those not moving to East village suggest similar associations between walkability and change in steps per day (Fig. 1). Pairwise correlations for change in walkability and change in distance to park (− 0.28), change in walkability and change in PTA (0.44), change in distance to park and change in PTAL (− 0.14) were not strong, suggesting that they measure distinctly different facets of the built environment.

It is thus apparent that changes in the measured built environment as predictors of change in physical activity are not the same as previously reported analyses comparing those who moved to East Village with those who did not. The following section therefore presents a more formal analysis of associations.

### Effect of the residential built environment on physical activity over time

Associations between changes in built environment factors and changes in total daily steps taken and daily amount of MVPA accumulated are presented in Table 3. *P*-values for the effect modification by housing groups, and effect sizes stratified by housing group are also shown in Table 3.

**Table 3:** Associations between increased walkability, greater distance to parks and increased accessibility to public transport and within-person change in daily steps and MVPA (min), examining effect modification by housing group (n = 687)

	Standardised effects ^a^		*P*-value for effect modification between housing groups	Standardised effects ^a^ for social housing seekers		Standardised effects ^a^ for intermediate housing seekers		Standardised effects ^a^ for market-rent housing seekers	
	β (95% CI)	*P*-value		β (95% CI)	*P*-value	β (95% CI)	*P*-value	β (95% CI)	*P*-value
Change in exposure (baseline to follow-up)	**Outcome: Daily steps**								
Walkability	302 (110;494)	0.002	0.48	129 (− 210;468)	0.46	380 (99;661)	0.008	392 (−24;808)	0.06
Connectivity	133 (−58;325)	0.17	0.79	65 (− 281;411)	0.71	126 (−149;401)	0.37	255 (− 172;683)	0.24
Residential density	313 (123;504)	0.001	0.92	306 (−5;616)	0.053	350 (66; 635)	0.02	237 (− 218;691)	0.31
Land use mix	201 (5;398)	0.04	0.49	51 (−264;366)	0.36	301 (17;584)	0.04	288 (− 244;808)	0.29
Distance to park	55 (− 136;247)	0.57	0.13	348 (−30;725)	0.07	−126 (− 397;144)	0.36	120 (− 266;507)	0.54
Access to public transport	−7 (− 205;191)	0.94	0.03	−295 (−595;3)	0.053	172 (− 122;466)	0.25	410 (−191;1010)	0.18
Change in exposure (baseline to follow-up)	**Outcome: Daily MVPA (min)**								
Walkability	1.7 (0.2;3.2)	0.03	0.27	0.0 (−2.6;2.7)	0.98	2.1 (−0.1;4.3)	0.06	3.4 (0.1;6.6)	0.04
Connectivity	1.1 (− 0.4;2.6)	0.14	0.70	0.0 (−2.8;2.9)	0.99	1.5 (−0.7;3.8)	0.19	1.5 (−2.0;5.0)	0.41
Residential density	1.7 (0.2;3.2)	0.03	0.79	0.9 (−1.6;3.5)	0.48	2.0 (−0.3;4.4)	0.09	0.9 (−2.9;4.6)	0.65
Land use mix	0.8 (−0.8;2.3)	0.34	0.58	−0.6 (−3.2;1.9)	0.62	1.0 (−1.4;3.3)	0.42	1.5 (−2.8;5.9)	0.49
Distance to park	0.6 (−0.9;2.1)	0.44	0.12	3.1 (0.2;6.1)	0.04	−0.7 (−2.8;1.4)	0.52	0.6 (−2.5;3.6)	0.72
Access to public transport	−0.2 (−1.8;1.3)	0.75	0.10	−1.8 (−4.2;0.5)	0.13	0.3 (−2.0;2.7)	0.76	3.6 (−1.1;8.3)	0.13

^a^ Size of effect are for 1 standard deviation (note that SD for changes in exposures are as follow: Walkability, 2.8; Residential density, 11.6; Land use mix, 0.26; Street connectivity, 1.3; Distance to park, 496; Access to public transport, 2.5)

Models are adjusted for sex, age group, ethnic group, housing group, and one of the “change in exposure” variables (entered in turn) as random effects and clustering at household level as the fixed effect. Further models with an interaction term between the “change in exposure” variable and housing group enabled the effect in each housing group to be calculated using linear combinations of the regression coefficients for the main and interaction terms in the model

In fully adjusted models, a 1 s.d. increase in neighbourhood walkability was associated with increases of 302 (95%CI 110 to 494) daily steps. For residential density, a 1 s.d. increase was associated with an increase in 313 (95%CI 123 to 504) daily steps. For land use mix, a 1 s.d. increase was associated with 201 (95%CI 5 to 398) more daily steps. These effects were consistent across housing groups, with no interaction terms reaching statistical significance.

Greater proximity to the nearest park at follow-up compared with baseline was not significantly associated with a change in any of the PA outcomes. These associations were not modified by housing group, with no interaction terms reaching statistical significance.

Increased accessibility to public transport was not significantly associated with a change in any of the PA outcomes in the whole sample. However, there was some evidence of interactions between accessibility to public transport and housing group in relation to mean daily steps were observed. A 1 s.d. increase in accessibility to public transport was borderline significant associated with a decrease in daily steps among social housing seekers (− 295 steps (95%CI -595, + 3), but, conversely, an increase in daily steps for market-rent housing seekers (410 95%CI -191, 1010) (*p*-value for effect modification of 0.03).

For completeness we assessed whether the statistically significant increase in steps remained after including other built environment variables (distance to closest park and PTAL) in the model (data not shown). In this fully adjusted model a 1 s.d. increase in neighbourhood walkability was associated with an increase of 412 steps (95% CI 194, 631 steps) compared to 302 steps (95% CI 110, 494 steps) in the model presented in Table 3. However, the regression coefficients for change in distance to park and change in PTAL are not statistically significant in this model. We therefore preferred the more conservative model presented in Table 3. Sensitivity analyses reported in Supplemental material 5 show that housing group differences in the association between increased accessibility to public transport and change in steps are greater on weekdays (*p*-value for effect modification of 0.007) compared with weekends (*p*-value for effect modification of 0.75). On weekdays, a 1 s.d. increase in accessibility to public transport was significantly associated with a 395 (95%CI − 720;-70) steps *decrease* among social housing seekers, but, conversely, a 657 (95%CI 4;1309) daily steps *increase* for market-rent housing seekers. On weekends, no such a pattern is observed.

## Discussion

At follow-up, study participants experienced positive changes in exposure to residential built environment factors hypothesised to support PA. Residential neighbourhood walkability improved, mainly through increases in residential density and land use mix. Participants also lived closer to their nearest park and had increased accessibility to public transport. Fully adjusted regression models indicated that a positive change in neighbourhood walkability was associated with a statistically significant increase in daily steps and daily amount of MVPA accumulated. These findings strengthen the evidence [6, 7, 24, 25] that more walkable environments are associated with higher levels of PA. These associations were mostly driven by two components of walkability: residential density and land use mix, which were both strongly and positively associated with increased PA levels. Greater land use mix is thought to support walking by offering greater accessibility to a wide range of services and employment, seen as potential walking destinations of interest [26]. For higher residential density, it is theorised to provide a critical mass of walkers seen by other people who may, in turn, be encouraged to walk by safety in numbers [27], and a desire to comply with the social norm of walking [28]. Traffic congestion associated with higher residential density may also promote more active modes of travel [29]. Unlike other studies [9, 10], we did not find evidence that changes in street connectivity were associated with a change in the number of steps taken or amount of MVPA accumulated. Since our street connectivity metric was derived from road network data only, it fell short in capturing pedestrianised areas and informal footpaths, which may be important contributors to the variety of routes in East Village. This may have had the effect of underestimating the magnitude of the association between street connectivity and PA.

We did not find evidence that increased accessibility to greenspace was associated with change in PA level at follow-up. This finding held true for the three housing groups. Previous research has highlighted the importance of disambiguating between different types of park, because park size and attractiveness influence their relation to PA [30], which may partly explain our null findings.

We also found weak evidence that having increased accessibility to public transport over time was associated with a decrease in PA among social housing seekers, but an increase in PA among market-rent housing seekers (the interaction was borderline significant). This suggests that only the more advantaged groups may benefit from policies aimed at increasing accessibility to public transport, possibly widening further socio-economic differences in PA levels. Our sensitivity analysis findings further show that such housing group differences were greater on weekdays compared with weekends, suggesting that these differences may be work related. The combined use of GPS and accelerometer data would be especially useful to further our understanding of this relationship.

Overall, our findings suggest that changing some elements of the residential built environment, especially neighbourhood walkability and to some extent accessibility to public transport, may impact PA levels. Despite the sizeable improvements in these built environment features associated with moving to East Village, Nightingale et al. [15] found that relocating to East Village did not translate into a commensurate increase in PA when compared to participants who did not move to East Village. The use of a dichotomous group-level exposure variable (movers to East Village vs non-movers to East Village) in Nightingale et al.‘s paper [15] may have reduced the ability to detect associations given the considerable individual-level variability in changes in environmental exposures within both the movers to East Village and the non-movers to East Village. These findings suggest that consideration of change in individual exposure to the built environment has greater power to demonstrate potentially positive effects on health behaviours, such as physical activity; however, to exploit this it is necessary to identify and measure the relevant exposure.

### Strengths

To our knowledge, this is the first study to have examined how changes in GIS-derived residential built environment features are associated with changes in objectively measured PA. It also enrolled a relatively high number of participants compared to other longitudinal studies (e.g. [10, 31]), increasing power. Moreover, the design of the ENABLE London study, by enrolling movers to East Village, movers to other neighbourhoods than East Village, as well as non-movers, provided us with considerable variability in the change of exposure, to different components of the built environment over time. Other strengths of this paper include the use of validated objective measures of PA [32], and exploring the contribution of the residential built environment in explaining socio-economic differences in PA levels.

### Limitations

Because the sample was not randomly selected, findings may not be generalizable to the broader population. Some aspects of the urban design assumed to promote PA behaviours (e.g. footpaths, pedestrianised areas) were not fully captured by traditional measures such as street connectivity. Residential selection cannot be completely dismissed from this study. This is where there is selective sorting into the East Village neighbourhood by those who favour, for example, neighbourhoods built with active design principles or who have higher underlying rates of physical activity. In addition, sample selection was based on respondents selection of their preferred residence,and as such, residential selection bias may be different to other studies. Changing our computed standard deviations into meaningful absolute changes in exposure is difficult. PTAL for example is a measure of the total density of connectivity of a transport network rather than a measure of a specific mode of transport. As such, this makes it useful for planning purposes, but does not give you a threshold to achieve*.* Finally, we were not able to align with calls for considering non-residential exposures to the built environment [33]. Not considering the built environment and PA facilities available in routinely visited settings other than place of residence (e.g. workplace) may have led to a misestimation of the association between the residential environment and health behaviours [34]. Combined use of GPS and GIS data would help move towards more context-specific measures of PA.

## Conclusion

Our findings suggest that changing some elements of the residential built environment may increase PA levels. We provide evidence for improving walkability, primarily driven through land-use mix and higher residential density, as a lever to increase adults PA. While the change in steps associated with an achievable 1 SD was modest, this could have worthwhile effects on mortality at a population level (i.e., 300 step difference could plausibly result in a 1–2% reduction in overall mortality) [35, 36]. However, for associations between accessibility to public transport and PA the findings were more nuanced. We observed that although there were increases in PA for more advantaged groups, there were decreases for more disadvantaged groups. This suggests a possible unintended consequence of improved access to public transport in widening inequalities in PA. Hence, interventions designed to improve the built environment to increase PA should carefully consider the potential for intervention-generated inequalities.

## Supplementary information

**Additional file 1.**

## Data Availability

The datasets used and/or analysed during the current study are available from the corresponding author on reasonable request. For general data sharing inquiries, contact Prof Owen (cowen@sgul.ac.uk).

## Abbreviations

ENABLE London: Examining Neighbourhood Activities in Built Living Environments in London
MVPA: Moderate to vigorous physical activity
PA: Physical activity
