# Supplementary material for: Longitudinal impact of changes in the residential built environment on physical activity: findings from the ENABLE London cohort study
Source: Int J Behav Nutr Phys Act. 2020 Aug 1;17:96. doi: 10.1186/s12966-020-01003-9 (PMC7395376; doi:10.1186/s12966-020-01003-9)
Supplement: Supplementary file 1 — Additional file 1. [file 12966_2020_1003_MOESM1_ESM.docx]

**Supplemental material 1** Definition, operationalisation and aggregation unit for the three residential built environment variables: street connectivity, residential density and land use mix

|  | **Definition** | **Operationalisation** | **Unit of aggregation** |
| --- | --- | --- | --- |
| Street connectivity | The directness and variety of routes to destinations | The number of 3 or more branch road junction per street kilometre | 1km-street network home-centred buffer |
| Residential density | The intensity with which land is occupied by either development or population | The number of residential units (i.e. unique households) per squared kilometre of land devoted to residential use (residential building footprint + attached gardens) | 1km-street network home-centred buffer |
| Land Use Mix | The degree of heterogeneity with which functionally different uses are co-located in space | The heterogeneity of distribution of square footage of residential, commercial, office, entertainment and institutional land.  $LUM=- \frac{\sum_{m=1}^{M} \pi_{m}\ln\pi_{m}}{\ln M}$  where  $\pi_{m}$ = the proportion of the area covered by the land use $m$ against the sum of the area of the *M* land uses of interest  *M* = the number of land use categories | 1km-street network home-centred buffer |

**Supplemental material 2** Data sources and versions used for computing the residential built environmental variables

|  | **BASELINE** | | | **FOLLOW-UP** | | |
| --- | --- | --- | --- | --- | --- | --- |
| Housing group | Social | Intermediate | Market-rent | Social | Intermediate | Market-rent |
|  |  |  |  |  |  |  |
| Median date of data collection | Aug 2013 | May 2014 | May 2015 | September 2015 | May 2016 | April 2017 |
|  |  |  |  |  |  |  |
| Street network data | ITN, version June 2015 | ITN, version June 2015 | ITN, version June 2015 | ITN, version June 2017 | ITN, version June 2017 | ITN, version June 2017 |
| Residential unit data | AddressBase Plus May, version 2013 | AddressBase Plus, version May 2014 | AddressBase Plus, version July 2015 | AddressBase Plus May, version June 2015 | AddressBase Plus, version June 2016 | AddressBase Plus, version May 2017 |
| Topography data | OS MasterMap Topography layer, version December 2013 | OS MasterMap Topography layer, version May 2014 | OS MasterMap Topography layer, version June 2015 | OS MasterMap Topography layer, version June 2015 | OS MasterMap Topography layer, version June 2016 | OS MasterMap Topography layer, version May 2017 |
| Park data | Greenspace information for Greater London (GiGL) data downloaded in 2015 | Greenspace information for Greater London (GiGL) data downloaded in 2015 | Greenspace information for Greater London (GiGL) data downloaded in 2015 | Greenspace information for Greater London (GiGL) data downloaded in 2015 | Greenspace information for Greater London (GiGL) data downloaded in 2015 | Greenspace information for Greater London (GiGL) data downloaded in 2015 |
| Transport for London (TfL) Public Transport Accessibility Levels (PTALs) | Version 2015 downloaded from the Datastore website* | Version 2015 downloaded from the Datastore website* | Version 2015 downloaded from the Datastore website* | Version 2015 downloaded from the Datastore website* | Version 2015 downloaded from the Datastore website* | Version 2015 downloaded from the Datastore website* |

* https://data.london.gov.uk/dataset/public-transport-accessibility-levels

**Supplemental material 3** Baseline sociodemographics of ENABLE London participants included in, and excluded from, the analyses

|  | Analytical sample (n=687) | |  | All excluded participants  (n=591) | | | | Excluded/Non-followed participants (n=401) | | | |  | Excluded/Followed participants with insufficient data (n=190) | | | |
| --- | --- | --- | --- | --- | --- | --- | --- | --- | --- | --- | --- | --- | --- | --- | --- | --- |
|  | n | % |  | n | % | Comparison with analytical sample | | n | % | Comparison with analytical sample | |  | n | % | Comparison with analytical sample | |
|  |  |  |  |  |  | %  point | Pearson Chi-square p-value |  |  | % point | Pearson Chi-square p-value |  |  |  | % point | Pearson Chi-square p-value |
| **Sociodemographics** |  |  |  |  |  |  |  |  |  |  |  |  |  |  |  |  |
| *Housing group* |  |  |  |  |  |  |  |  |  |  |  |  |  |  |  |  |
| Social | 283 | 41.2 |  | 237 | 40.1 | -1.1 | 0.003 | 176 | 43.9 | +2.7 | 0.04 |  | 61 | 32.1 | -9.1 | <0.001 |
| Intermediate | 301 | 43.8 |  | 223 | 37.7 | -6.1 |  | 147 | 36.6 | -7.2 |  |  | 76 | 40.0 | -3.8 |  |
| Market-rent | 103 | 15.0 |  | 131 | 22.2 | +7.2 |  | 78 | 19.5 | +4.5 |  |  | 53 | 27.9 | +12.9 |  |
| *Sex* |  |  |  |  |  |  |  |  |  |  |  |  |  |  |  |  |
| Female | 401 | 58.4 |  | 330 | 55.8 | -2.6 | 0.36 | 236 | 58.9 | +0.5 | 0.88 |  | 94 | 49.5 | -8.9 | 0.03 |
| *Age groups* |  |  |  |  |  |  |  |  |  |  |  |  |  |  |  |  |
| 16-24 years | 130 | 18.8 |  | 145 | 24.5 | +5.7 | 0.008 | 96 | 23.9 | +5.1 | 0.10 |  | 49 | 25.8 | +7.0 | 0.01 |
| 25-34 years | 289 | 42.1 |  | 259 | 43.8 | +1.7 |  | 170 | 42.5 | +0.4 |  |  | 89 | 46.8 | +4.7 |  |
| 35-49 years | 223 | 32.5 |  | 145 | 24.5 | -8.0 |  | 106 | 26.4 | -6.1 |  |  | 39 | 20.6 | -11.9 |  |
| 50+ years | 45 | 6.6 |  | 42 | 7.2 | +0.6 |  | 29 | 7.2 | +0.6 |  |  | 13 | 6.8 | +0.2 |  |
| *Ethnicity* |  |  |  |  |  |  |  |  |  |  |  |  |  |  |  |  |
| White | 334 | 48.6 |  | 283 | 47.9 | -0.7 | 0.76 | 180 | 44.9 | -3.7 | 0.67 |  | 103 | 54.1 | +5.5 | 0.07 |
| Black | 172 | 25.0 |  | 151 | 25.5 | +0.5 |  | 111 | 27.7 | +2.7 |  |  | 40 | 21.1 | -3.9 |  |
| Asian | 110 | 16.0 |  | 104 | 17.6 | +1.6 |  | 67 | 16.7 | +0.7 |  |  | 37 | 19.5 | +3.5 |  |
| Mixed/Other | 71 | 10.4 |  | 53 | 9.0 | -1.4 |  | 43 | 10.7 | +0.3 |  |  | 10 | 5.3 | -5.1 |  |

**Supplemental material 4** Within-person change (baseline to follow-up) in residential built environment characteristics by aspirational housing tenure and combined (n=687), for the movers to East Village, and the non-movers to East Village (including those who relocated elsewhere in London and those who remained at the same address)

|  | **Total** | | |  | **Housing group** | | | | | | | | | | | | |  | |
| --- | --- | --- | --- | --- | --- | --- | --- | --- | --- | --- | --- | --- | --- | --- | --- | --- | --- | --- | --- |
| **Movers to East Village** | **n=357** | | |  | **Social n=183** | | | | **Intermediate n=142** | | | | **Market-rent n=32** | | | | | **p-value ^a^** | |
| *PA facilities in the residential* *area* | Mean | (95%CI) | |  | mean | | (95%CI) | | mean | | (95%CI) | | | mean | | (95%CI) | |  | |
| Change in walkability (score) | 2.4 | (2.1;2.7) | |  | 2.7 | | (2.4;3.0) | | 2.1 | | (1.6;2.6) | | | 2.3 | | (0.5;4.1) | | 0.13 | |
| Change in street connectivity (nb intersections/km of road) | -1.0 | (-1.1;-0.8) | |  | -0.8 | | (-1.0;-0.6) | | -1.1 | | (-1.4;-0.9) | | | -1.1 | | (-1.8;-0.3) | | 0.05 | |
| Change in residential density (1000hab/km2) | 13.3 | (12.0;14.6) | |  | 12.9 | | (11.3;14.6) | | 12.9 | | (10.8;15.0) | | | 17.3 | | (11.5;23.0) | | 0.18 | |
| Change in Land Use Mix (score) | 0.38 | (0.36;0.40) | |  | 0.39 | | (0.36;0.41) | | 0.38 | | (0.34;0.41) | | | 0.33 | | (0.22;0.43) | | 0.34 | |
| Change in distance to the closest park (km) | -531 | (-574;-488) | |  | -465 | | (-516;-413) | | -583 | | (-651;-515) | | | -680 | | (-907;-453) | | 0.008 | |
| Change in accessibility to public transport (PTAL score) | 1.6 | (1.3;1.9) | |  | 2.5 | | (2.1;2.8) | | 0.7 | | (0.3;1.2) | | | 0.5 | | (-0.5;1.5) | | <0.001 | |
| ^a^ anova |  |  | |  |  | |  | |  | |  | | |  | |  | |  | |
|  | **Total** | |  | **Housing group** | | | | | | | | | | | | |  | |  |
| **Non-movers to East Village** | **n=330** | |  | **Social  n=100** | | | | **Intermediate  n=159** | | | | **Market-rent n=71** | | | | | **p-value ^a^** | |  |
| *PA facilities in the residential* *area* | mean | (95%CI) |  | mean | | (95%CI) | | mean | | (95%CI) | | mean | | | (95%CI) | |  | |  |
| Change in walkability (score) | 0.3 | (0.1;0.6) |  | -0.1 | | (-0.5;0.3) | | 0.5 | | (0.1;0.9) | | 0.4 | | | (-0.1;0.9) | | 0.14 | |  |
| Change in street connectivity (nb intersections/km of road) | 0.0 | (-0.1;0.1) |  | -0.1 | | (-0.3;0.1) | | 0.1 | | (-0.1;0.2) | | -0.1 | | | (-0.3;0.2) | | 0.47 | |  |
| Change in residential density (1000hab/km2) | 1.9 | (1.1;2.6) |  | 1.6 | | (0.7;2.4) | | 2.1 | | (0.9;3.3) | | 1.8 | | | (0.1;3.4) | | 0.82 | |  |
| Change in Land Use Mix (score) | 0.02 | (0.00;0.04) |  | -0.02 | | (-0.05;0.01) | | 0.04 | | (0.01;0.07) | | 0.03 | | | (-0.01;0.06) | | 0.03 | |  |
| Change in distance to the closest park (km) | -13 | (-32;59) |  | -6 | | (-62;50) | | 24 | | (-45;94) | | 15 | | | (-106;136) | | 0.85 | |  |
| Change in accessibility to public transport (PTAL score) | -0.2 | (-0.4;0.0) |  | -0.2 | | (-0.5;0.0) | | -0.2 | | (-0.5;0.1) | | 0.0 | | | (-0.4;0.4) | | 0.78 | |  |
| ^a^ anova |  |  |  |  | |  | |  | |  | |  | | |  | |  | |  |
|  | **Total** | | |  | **Housing group** | | | | | | | | | | | | |  | |
| **Non-movers to East Village, who relocated elsewhere in London** | **n=161** | | |  | **Social  n=31** | | | | **Intermediate  n=92** | | | | | **Market-rent n=38** | | | | **p-value ^a^** | |
| *PA facilities in the residential* *area* | mean | (95%CI) | |  | mean | | (95%CI) | | mean | | (95%CI) | | | mean | | (95%CI) | |  | |
| Change in walkability (score) | 0.0 | (-0.5;0.5) | |  | -0.7 | | (-1.9;0.5) | | 0.3 | | (-0.4;1.0) | | | 0.0 | | (-0.9;1.0) | | 0.36 | |
| Change in street connectivity (nb intersections/km of road) | -0.1 | (-0.4;0.1) | |  | -0.4 | | (-1.0;0.1) | | 0.0 | | (-0.3;0.3) | | | -0.3 | | (-0.7;0.2) | | 0.34 | |
| Change in residential density (1000hab/km2) | 1.2 | (0;2.6) | |  | 0.5 | | (-2.1;3.0) | | 1.4 | | (-0.6;3.4) | | | 1.4 | | (-1.6;4.4) | | 0.88 | |
| Change in Land Use Mix (score) | 0.01 | (-0.03;0.05) | |  | -0.04 | | (-0.13;0.06) | | 0.03 | | (-0.03;0.08) | | | 0.01 | | (-0.06;0.07) | | 0.48 | |
| Change in distance to the closest park (km) | 27 | (-66;120) | |  | -15 | | (-203;174) | | 43 | | (-78;164) | | | 24 | | (-208;255) | | 0.90 | |
| Change in accessibility to public transport (PTAL score) | -0.3 | (-0.7;0.1) | |  | -0.7 | | (-1.6;0.2) | | -0.3 | | (-0.8;0.2) | | | -0.1 | | (-0.9;0.7) | | 0.55 | |
|  |  |  | |  |  | |  | |  | |  | | |  | |  | |  | |
|  |  |  | |  |  | |  | |  | |  | | |  | |  | |  | |
|  | **Total** | | |  | **Housing group** | | | | | | | | | | | | |  | |
| **Non-movers to East Village, who remained at the same address** | **n=169** | | |  | **Social  n=69** | | | | **Intermediate  n=67** | | | | | **Market-rent n=33** | | | | **p-value ^a^** | |
| *PA facilities in the residential* *area* | mean | (95%CI) | |  | mean | | (95%CI) | | mean | | (95%CI) | | | mean | | (95%CI) | |  | |
| Change in walkability (score) | 0.6 | (0.5;0.6) | |  | 0.2 | | (0.1;0.3) | | 0.8 | | (0.7;0.9) | | | 0.8 | | (0.6;0.9) | | <0.001 | |
| Change in street connectivity (nb intersections/km of road) | 0.1 | (0.1;0.1) | |  | 0.1 | | (0.0;0.1) | | 0.1 | | (0.1;0.2) | | | 0.1 | | (0.0;0.2) | | 0.15 | |
| Change in residential density (1000hab/km2) | 2.5 | (2.1;2.9) | |  | 2.1 | | (1.6;2.5) | | 3.0 | | (2.2;3.9) | | | 2.2 | | (1.4;3.0) | | 0.09 | |
| Change in Land Use Mix (score) | 0.03 | (0.02;0.04) | |  | -0.01 | | (-0.02;0.0) | | 0.06 | | (0.05;0.07) | | | 0.05 | | (0.04;0.06) | | <0.001 | |
| Change in distance to the closest park (km) | 0.0 | (-2;2) | |  | -2 | | (-5;1) | | -1 | | (-2;1) | | | 5 | | (-2;11) | | 0.059 | |
| Change in accessibility to public transport (PTAL score) | 0.0 | (0.0;0.0) | |  | 0.0 | | (0.0;0.0) | | 0.0 | | (-0.1;0.0) | | | 0.0 | | (0.0;0.0) | | 0.47 | |

**Supplemental material 5** Associations between within-person change in daily steps and MVPA (min) and increased accessibility to public transport on weekdays and on weekends, and effect modification by housing group

|  |  | Standardised effects ^a^ | | P-value for effect modification between housing groups |  | Standardised effects ^a^ for **social housing seekers** | | | Standardised effects ^a^ for **intermediate housing seekers** | | Standardised effects ^a^ for **market-rent housing seekers** | |  |
| --- | --- | --- | --- | --- | --- | --- | --- | --- | --- | --- | --- | --- | --- |
|  |  |  |  |  |  |  |  |  |  |  |  |  |  |
|  |  | β (95% CI) | P-value |  |  | β (95% CI) | P-value | β (95% CI) | | P-value | β (95% CI) | P-value | |
|  |  | **Outcome: Daily steps on weekdays (n=684)** | | | | | | | | | | | |
| **WEEKDAYS** | Change in access to public transport | -44 (-259;172) | 0.69 | 0.007 |  | -395 (-720;-70) | 0.02 | 124 (-194;443) | | 0.44 | 657 (4;1309) | 0.049 | |
|  |  | **Outcome: Daily MVPA (min) on weekdays (n=684)** | | | | | | | | | | | |
|  | Access to public transport | -0.7 (-2.5;1.0) | 0.40 | 0.01 |  | -2.9 (-5.5;-0.3) | 0.03 | -0.2 (-2.8;2.3) | | 0.87 | 5.7 (0.4;10.9) | 0.03 | |
|  |  | **Outcome: Daily steps on weekends (n=517)** | | | | | | | | | | | |
| **WEEKENDS** | Change in access to public transport | 12 (-348;373) | 0.95 | 0.75 |  | -32 (-262;198) | 0.78 | 60 (-149;269) | | 0.57 | -103 (-548;343) | 0.65 | |
|  |  | **Outcome: Daily MVPA (min) on weekends (n=517)** | | | | | | | | | | | |
|  | Access to public transport | 0.1 (-2.7;2.9) | 0.94 | 0.83 |  | 0.0 (-1.9;1.8) | 0.95 | 0.3 (-1.3;2.0) | | 0.7 | -0.8 (-4.4;2.7) | 0.63 | |

^a^ Size of effect are for 1 standard deviation (note that SD for changes in exposures are as follow: Walkability, 2.8; Residential density, 11.6; Land use mix, 0.26; Street connectivity, 1.3; Distance to park, 496; Access to public transport, 2.5)

Models are adjusted for sex, age group, ethnic group, aspirational housing tenure, clustering at household level, and one of the “change in exposure” variables (entered in turn)

*Note: Mean daily steps on weekdays were derived as follows. First, a multilevel linear model was fitted to allow for repeated measurements of daily weekday steps, by fitting participant as a random effect and adjusting for day of the week, day order of recording, and month as fixed effects. Second, raw level one residuals were obtained from the model, and a within-person average value was obtained by averaging these raw residuals. Third, the average of these raw residuals for each participant was added to the sample mean of steps on weekdays to derive an average level of daily steps on weekday for each person.*

*A similar process was followed to derive mean daily steps on weekends, mean MVPA on weekdays, and mean MVPA on weekends.*

**Supplemental material 6** Mean and 95% confidence intervals for change in daily steps by fourths of the distribution of change in walkability.

***East Village and Control groups combined***

**

***East Village and Control groups separately***

**
